# Understanding Information Needs and Barriers to Accessing Health Information Across All Stages of Pregnancy: Systematic Review

**DOI:** 10.2196/32235

**Published:** 2022-02-21

**Authors:** Yu Lu, Laura A Barrett, Rebecca Z Lin, Muhammad Amith, Cui Tao, Zhe He

**Affiliations:** 1 School of Information Florida State University Tallahassee, FL United States; 2 Washington University School of Medicine at St. Louis St. Louis, MO United States; 3 School of Biomedical Informatics University of Texas Health Science Center at Houston Houston, TX United States

**Keywords:** pregnancy, information needs, ontology, systematic review, fertility, parenting, pregnancy information, online information, health database

## Abstract

**Background:**

Understanding consumers’ health information needs across all stages of the pregnancy trajectory is crucial to the development of mechanisms that allow them to retrieve high-quality, customized, and layperson-friendly health information.

**Objective:**

The objective of this study was to identify research gaps in pregnancy-related consumer information needs and available information from different sources.

**Methods:**

We conducted a systematic review of CINAHL, Cochrane, PubMed, and Web of Science for relevant articles that were published from 2009 to 2019. The quality of the included articles was assessed using the Critical Appraisal Skills Program. A descriptive data analysis was performed on these articles. Based on the review result, we developed the Pregnancy Information Needs Ontology (PINO) and made it publicly available in GitHub and BioPortal.

**Results:**

A total of 33 articles from 9 countries met the inclusion criteria for this review, of which the majority were published no earlier than 2016. Most studies were either descriptive (9/33, 27%), interviews (7/33, 21%), or surveys/questionnaires (7/33, 21%); 20 articles mentioned consumers’ pregnancy-related information needs. Half (9/18, 50%) of the human-subject studies were conducted in the United States. More than a third (13/33, 39%) of all studies focused on during-pregnancy stage; only one study (1/33, 3%) was about all stages of pregnancy. The most frequent consumer information needs were related to labor delivery (9/20, 45%), medication in pregnancy (6/20, 30%), newborn care (5/20, 25%), and lab tests (6/20, 30%). The most frequently available source of information was the internet (15/24, 63%). PINO consists of 267 classes, 555 axioms, and 271 subclass relationships.

**Conclusions:**

Only a few articles assessed the barriers to access to pregnancy-related information and the quality of each source of information; further work is needed. Future work is also needed to address the gaps between the information needed and the information available.

## Introduction

As a widely discussed topic in women’s health, pregnancy is an important phase of women’s lives, a period in which women experience biological changes and gain a new identity at the same time [1]. Importantly, pregnancy is often accompanied by various complications. According to the Blue Cross Blue Shield Association [2], pregnancy complications occurred in 1 in 5 pregnancies among prospective mothers aged 18 to 44 years (2014-2018). Even though regular medical monitoring and prenatal testing are essential to ensure healthy pregnancy, they may provoke anxiety, especially for those who experience complications in their pregnancy. Besides pregnancy, infertility presents a concerning issue since 1 in 8 couples have experienced fertility problems [3]. The Centers of Disease Control and Prevention reported that about 6% of married women aged 15 to 44 years in the United States experience infertility after 1 year of trying [4]. The internet has been an important source of information that can help women deal with doubts and make pregnancy-related decisions [5]. According to a nationwide survey in the United States, more than 75% of childbearing women searched for information related to pregnancy and childbirth on the internet [6]. However, resources related to pregnancy are often scattered, conflicting, and hard to appraise and understand [7]. It was found that patients with limited health literacy often have difficulties finding useful medical information online that is contextualized to their conditions [8]. The emergence of Web 2.0 health technologies, such as blogs, smartphone apps, and online health communities, provides ways for pregnant women to proactively interact with the community by posting questions with detailed information, sharing their experiences, and providing answers to others pregnancy-related questions [9,10].

Previously, researchers have attempted to understand the consumer information needs related to pregnancy and infertility [11,12]. Moreover, systematic reviews have assessed the use of the internet, health information needs, sources of information, and barriers to accessing health information among pregnant women [13,14]. For example, Sayakhot and Carolan-Olah [14] reported that pregnant women often search the internet for different topics such as medication, nutrition, and fetal development during their pregnancy. Ghiasi [13] found that women expressed various information needs during their pregnancy. However, these reviews mostly focused on a certain stage of pregnancy. In reality, consumer information needs across different stages are correlated with each other and certain needs may span all stages of pregnancy. Hence, a study that systematically organizes pregnancy-related information is necessary yet unavailable to date. Such a study would allow us to better understand the consumer information needs across the span of pregnancy and find opportunities to better meet these needs.

To fill this gap, we performed a systematic review of the published literature related to consumer information needs and sources across all stages of pregnancy: including prepregnancy, which refers to the stage prior to pregnancy [15]; pregnancy, the condition of being pregnant [15]; and postpartum, the period following childbirth [16]. To support subsequent development of pregnancy apps such as websites, mobile apps, and patient portals, we also created a taxonomy of pregnancy information. This has helped us to identify a number of important research gaps and opportunities.

## Methods

### Literature Search Strategy

In this study, following the Preferred Reporting Items for Systematic Review and Meta-analysis (PRISMA) guideline [17], we performed a systematic review of the literature regarding pregnancy-related consumer information needs from 2009 to 2019 from 4 major databases: CINAHL, Cochrane Reviews, PubMed, and Web of Science using the search queries “health information” and (pregnan* or fertility or infertility or conception or mother* or matern* or prenat* or pre-nat* or antenat* or ante-nat* or perinat* or peri-nat* or pre-pregnancy or pre-pregna* or gestation*).

**Figure 1:**
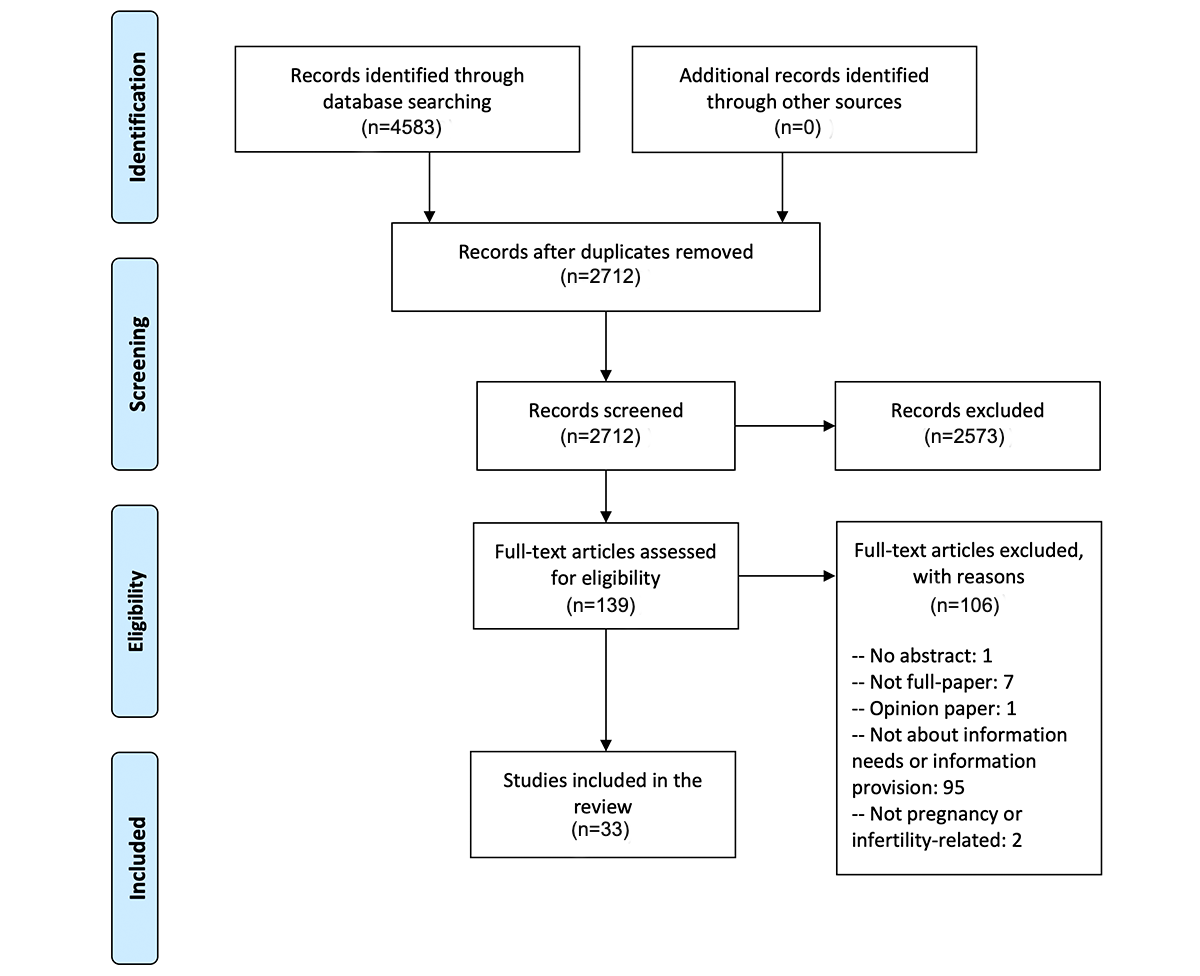
PRISMA flowchart of paper selection process.

In total, we found 4583 articles. After removing duplicates, 2712 articles remained. We excluded 2573 articles after the title and abstract screening. Then we performed full-text review on the remaining 139 articles, including articles about pregnancy information needs or sources, pregnancy- or infertility-related, that were published in English from 2009 to 2019 and excluding articles that were not full papers, were opinion papers, or did not contain as abstract. The remaining 33 articles were evaluated using the Critical Appraisal Skills Program (CASP) [18] for quality assurance and were determined to have adequate quality to be included in the final full-text extraction. The detailed information about CASP review can be found in the Multimedia Appendix 1. The PRISMA workflow is shown in Figure 1.

### Data Extraction

In the evaluation of the full text of the 33 articles, we identified the following aspects of information: (1) year when the article was published, (2) topic of the study, (3) research method of the study, (4) sample size of the study (if human subjects were used), (5) sources of the health information, (6) stages of pregnancy on which the study focused, (7) target population, (8) country, and (9) consumer information needs.

## Results

### Overall Trends

**Figure 2:**
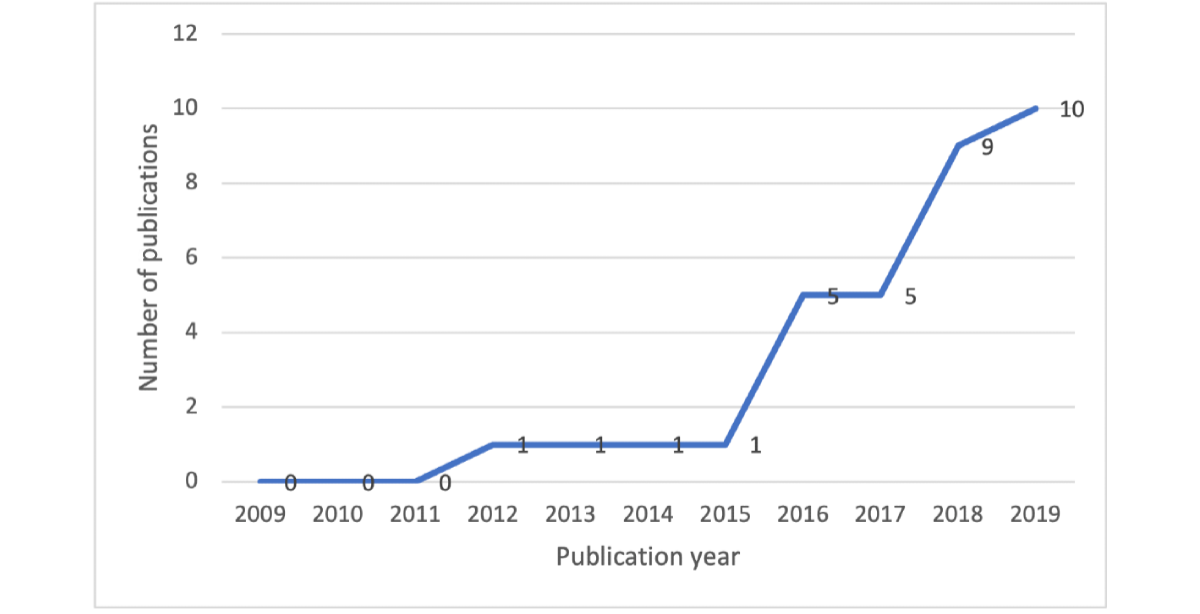
Number of extracted papers in each year (2009-2019).

The trend of the number of the included articles is shown in Figure 2. Between 2009 and 2015, there was a small number of studies about pregnancy-related consumer information needs and sources. Since 2015, there has been a surge in the number of studies published.

### Methods, Sample Sizes, Health Information Sources, and the Stages of Pregnancy

#### Characteristics of Included Papers

The characteristics (eg, topic, study type, sample size, health information source, stage of pregnancy, and target population) of all included studies are presented in Multimedia Appendix 2.

#### Methods Used for Data Collection in Included Studies

Among all methods, secondary data (11/33, 33%), interview (7/33, 21%), and survey/questionnaire (8/33, 24%) were the most frequently adopted methods, followed by systematic review (4/33, 12%), participatory design (1/33, 3%), randomized controlled trials (1/33, 3%), and focus group (1/33, 3%). We present these data collection methods as follows.

#### Secondary Data

Studies that leveraged secondary data assessed various population groups’ consumer information needs about different aspects of pregnancy. For instance, Kallem et al [19] investigated new mothers’ information needs related to newborn health. The results suggested that sleep and the appropriate use of screen time or media for infants were 2 topics that women commonly posted practices that were inconsistent with expert recommendations. Holton et al [20] analyzed group discussions of women with polycystic ovary syndrome on Facebook to assess their fertility-related consumer information needs. The results suggested that evidence-based information in various formats (eg, fact sheets, trusted websites, and podcasts) could help women with polycystic ovary syndrome make informed decisions about childbearing and achieve their reproductive goals. Van De Belt et al [12] analyzed questions in online forums and phone consultations to examine the gaps in information provision to infertile Dutch patients. The results showed that infertile patients demand high-quality health information and the information from health care providers did not cover all reported consumer information needs. Hence providers should explore new means of health information creation that involve the patient perspective [12].

#### Interview

Among the interview studies, Rotich and Wolvaardt [21] interviewed 15 pregnant women to assess Kenyan women’s pregnancy-related consumer information needs. The results suggested that pregnant women sought information about the expected changes after delivery and health care providers did not provide enough explanations of routine activities for newborns (eg, drawing blood from babies). Owusu-Addo et al [22] performed interviews to assess the information-seeking behavior of pregnant teenagers in rural Ghana. The interviews revealed that participants generally relied on traditional sources, such as family and neighbors; thus, health promotion interventions should target both the expecting teenagers and the family/community. Zhu et al [10] interviewed 20 Chinese women who had conceived and were currently in any stage of pregnancy. The interviews were recorded and used in a thematic analysis to identify major themes of participants’ information seeking and sharing via social media. The results suggested that participants’ information needs spanned all stages of pregnancy (prepregnancy, during pregnancy, and postpartum) and most of them were moderately or highly satisfied with the current provision of pregnancy-related information. Pang et al [23] conducted semistructured interviews to investigate online health information-seeking behavior among women who had experienced miscarriage. The results demonstrated that women sought information about miscarriage, miscarriage prevention, and current research advances, along with online support through peer experience and support from family and friends.

#### Survey/Questionnaire

Among the survey studies, Song et al [24] conducted a survey to assess consumer information needs, information-seeking behavior, and family support among low-income expectant mothers. The results indicated that information obtained from family can lead to the most positive difference in supporting women who are expecting or preparing to have a baby. Ceulemans et al [25] conducted a survey in Belgium to assess pregnant women’s information needs and beliefs about medications in general. The results suggested that pregnant women generally showed positive attitudes toward medications and high education levels suggest high thresholds to use medications during pregnancy. Cramer [26] performed a survey study to investigate expectant fathers’ health information-seeking behavior during pregnancy. The results suggested that paternal information needs were diverse and could change across stages of child development, interpersonal sources of information were important both before and after childbirth, and a close relationship between the expectant/new father and his partner is the key predictor of paternal health information seeking. Some studies used both survey and interview to assess pregnancy-related needs. For instance, Robinson et al [11] surveyed pregnant women and their caregivers about their demographic information; and then conducted interviews with the participants about their pregnancy-related consumer information needs. The results of the study suggested that consumers required pregnancy-related information about prognosis, health management, tests, interventions, logistics, and psychological support. Guerra-Reyes et al [27] used a survey and interviews to understand the postpartum health information-seeking behavior of low-income women using mobile apps. They found that mobile apps were used mostly during pregnancy but not postpartum, although low-income postpartum women do rely on mobile apps for infant care and personal health information.

#### Systematic Review of Websites, Apps, and Papers

Cannon et al [28] assessed pregnancy-related information on nutrition and physical activity websites and found that the nutrition-related information provided by those websites did not align with the guidelines. Brown et al [29] assessed nutrition-related information provided by mobile apps to pregnant women and found that these apps provided information about topics such as food safety, alcohol consumption, seafood consumption, caffeine consumption, and the recommended number of daily servings from key food groups for pregnant women. Ghiasi [13] conducted a systematic review of published papers to assess the health information needs of pregnant women and found that they often sought information about prenatal care, managing discomforts, environmental cleanliness, personal hygiene, sexual activity during pregnancy, medicine use, nutrition, and the development of fetus. Postpartum women often searched for information related to self-care after childbirth, breastfeeding, physical and mental complications after childbirth, newborn care, and family planning.

#### Focus Group

Arcia et al [30] conducted a focus group study to assess how low-income pregnant women characterize their information needs and found that this population’s needs span a wide range of topics, including pregnancy discomforts, environmental exposures, cloth diapering, and treating anemia.

#### Participatory Design

Linden et al [31] performed a participatory design and evaluation to assess the web-based provision of information to pregnant women with diabetes. The design proved to be a functional way of creating appropriate health information for the target group.

#### Randomized Controlled Trial

Kallem et al [19] conducted a randomized controlled trial to assess low-income urban mothers’ Facebook posts about infant health. They found that peers’ answers to mothers’ question posts generally did not contradict with the American Academy of Pediatrics guidelines.

#### Countries From Which Participants Were Recruited

Half of the studies which recruited human subjects recruited participants in the United States (9), followed by Australia (2), and 1 each from Canada, Belgium, China, Ghana, Iran, Kenya, and the Netherlands.

#### Sample Size

Over half of all human-subject studies had a sample size less than 50 (10/19, 53%), following by sample size of 51-100 (2/19, 11%), and 351-400 (2/19, 11%). Only a few studies (4/19) had a sample size over 400. We summarize the findings of the studies with over 100 participants as follows. Narasimhulu et al [32] conducted a cross-sectional study of 503 pregnant/postpartum women to assess their patterns of eHealth use. The findings implied that pregnant women frequently use eHealth resources but do not routinely share their findings with their providers. Kamali et al [33] performed a descriptive study on 400 women to assess their consumer information needs during pregnancy and childbirth. The main finding was that most women searched for information when they are suffering from a disease or pregnancy complication. Cramer [26] surveyed 186 expectant and recent fathers to investigate their health information behavior during pregnancy. The results suggested that paternal information needs were diverse and could change across stages of child development. Kriss et al [34] surveyed a total of 486 pregnant women to assess disparities in Tdap vaccination (tetanus toxoid, reduced diphtheria toxoid, and acellular pertussis) among pregnant women in the United States. They found that provider recommendation was one of the most important factors in Tdap vaccination. Brochu et al [35] surveyed 567 participants (men and women) to determine whether web-based sources met their consumer information needs related to infertility. The results implied that certain infertility-related consumer information needs were not always met and that patients may benefit form alternative sources. Ceulemans et al [25] investigated beliefs and consumer information needs about medicines among 372 pregnant women in Belgium. The results suggested that providers should be aware of pregnant women’s beliefs about medicine and guide them toward reliable sources.

#### Stages of Pregnancy

A large proportion of the included articles only focused on during pregnancy (13/33, 39%), followed by during pregnancy and postpartum (8/33, 24%) and postpartum only (7/33, 21%). Other studies also assessed consumer information needs only at the prepregnancy stage (4/33, 12%), and across all stages (1/33, 3%).

#### Consumer Information Needs

Out of the 33 articles, 20 mentioned pregnancy-related information. During prepregnancy stage, the most frequently raised topics was infertility (3/20,15%), such as the cause of infertility and treatment information and options. During pregnancy, the most frequently mentioned consumer information needs included lifestyle in pregnancy (9/20, 45%), such as nutrition for pregnant women and daily activities in pregnancy; labor/delivery (9/20, 45%), such as labor analgesia and labor pain and relief methods; prenatal care (8/20, 40%), such as the development and safety of the fetus; medication during pregnancy (6/20, 30%), such as the safety of medications; and vaccination during pregnancy (5/20, 25%), such as side effects of vaccines. At the postpartum stage, information about newborn care (5/20, 25%) was frequently sought, such as neonatal complications and newborn feeding. Across all stages of pregnancy, consumers often sought information about lab tests (6/20, 30%; eg, interpreting test results and DNA testing); and mental health (4/20, 20%; eg, preexisting or postpartum anxiety or depression and stress management).

#### Health Information Sources

We analyzed the percentage of health information sources in all applicable included studies (24). The most frequent health information source is the internet (15/24, 63%), followed by mobile apps (3/24, 13%), and health care providers (2/24, 8%). Some studies (2/24, 8%) used both the internet and health care providers as information sources. Another study mentioned using health care providers, family and friends, and the internet as sources (2/24, 8%). Some papers in this review described the sources of information they used [36,37]. For example, the World Health Organization [36], National Health and Medical Research Council of Australia [36], United Nations Children’s Fund [36], Australian Society of Clinical Immunology and Allergy [36], AmericanPregnancy.org [37], WebMD [37], BabyCenter.com [37], BreastfeedingUSA.org [37], NHS.uk [37].

#### Types of Pregnancy-Related Information Available in Each Source

We noticed that although all sources of information (including the internet, mobile apps, and health care providers) provided information related to pregnancy symptoms [33,38], nutrition and physical activity [28,29,31,33,38], labor [31,33,38], and postpartum care [33,37,38]. However, each of these sources also provided various types of information about pregnancy that were not available in the other 2 sources. The internet often provided information related to preconception [37], fertility preservation options [39], maternal diet [36], sleep [28], and congenital heart defects [40]. Mobile apps often provided information related to body changes during pregnancy [38], weight gain [38], finding a doctor or midwife [38], planning for a newborn [38], and mental health [27]. Health care providers often provided information related to body changes during pregnancy [33], vaccines [33], tests [33], prenatal education [33], medication [12], infertility treatment [12], abortion [33], personal hygiene [33], sexual activity in pregnancy [33], newborn feeding [33], newborn care [33], and physical/psychological complications after delivery [33].

### Barriers to Access

Two articles pointed out that consumers face certain barriers to accessing pregnancy-related information [13,33]. Kamali et al [33] found that lack of knowledge and ignorance regarding existing resources are 2 frequent barriers to accessing health information among pregnant women. In addition, the systematic literature review by Ghiasi [13] classified the barriers to accessing health information into 7 categories: (1) affective barrier included feeling ashamed or embarrassed to talk about pregnancy-related issues; (2) cognitive barrier included negative attitudes of health care providers, lack of familiarity with the internet for information seeking, poor patient-provider communication, reliance on self, underestimation of the risks of pregnancy complications, and unawareness of relevant information sources; (3) cultural barrier mainly referred to the social stigma related to pregnancy; (4) availability barrier included long wait times to see a doctor in clinic, lack of adequate information sources, and inadequate information from health care providers; (5) accessibility barrier referred to lack of time; (6) affordability barrier included high cost of information sources; and (7) infrastructure barrier referred to erratic power supply to access electronic information.

In addition, as shown in the reviewed papers, there were 3 scenarios regarding the accessibility of information: (1) needed information does not exist [12,41], (2) needed information exists but is not easily accessible (eg, consumers do not know how to find it or it is not easily readable) [32,39,42], and (3) needed information exists but in an incomprehensive or inconsistent manner [12].

### Quality of Information

Several studies also assessed the quality of information provided by these sources. For example, Brown et al [29] investigated the quality of information provided by iPhone apps for pregnancy and nutrition using the Mobile Application Rating Scale tool; the Coventry, Aberdeen and London–Refined taxonomy; and expert review. The results showed that the included apps were generally of moderate quality. De Man et al [39] assessed the availability and quality of online health information about fertility in the cancer setting using the DISCERN instrument, the Minervation validation instrument for health care websites, and a readability test. The results suggested that the included websites had moderate scores in readability and usability. However, the readability test did not consider medical jargon. Carlsson et al [40] assessed the quality of online information related to congenital heart defect following a prenatal diagnosis. The results showed that the majority of included websites scored poor in quality and readability with respect to the various themes about congenital heart defects.

### Pregnancy Information Needs Ontology

A secondary contribution of this study is an ontology about pregnancy information needs that contains information across all stages of pregnancy (prepregnancy, during pregnancy, and postpartum). To develop the preliminary version of Pregnancy Information Needs Ontology (PINO), one researcher (YL) first enumerated important terms about pregnancy information needs based on all reviewed papers. The goal of the enumeration was to obtain a comprehensive list of the terms. As certain terms may overlap with others in the meaning they represent, the researcher then synthesized terms that convey similar meanings. In the next step, the researcher developed a class hierarchy using a top-down approach, with the resulting hierarchy organizing information into themes and subnodes. The last step defined the properties of terms. PINO consists of 267 classes, 555 axioms, and 271 subclass relationships. In future work, we will evaluate both the intrinsic aspects (eg, concept orientation, consistency, soundness) and extrinsic aspects with the support of tools [43,44]. Table 1,2, 3, and 4 present subtaxonomies of PINO for each stage of the pregnancy trajectory, including prepregnancy, during pregnancy, postpartum, and across all stages.

**Table 1:** Taxonomy of pregnancy-related information: prepregnancy stage.

Theme and subnodes		N	Source
Preconception		1	[37]
Confirming pregnancy		1	[22]
Foster parenting		1	[35]
**Infertility**		3	[10,35,41]
	Causes of infertility	1	[10]
	Treatment information/options	1	[35]
	Diagnosis of infertility	1	[35]
	Medications used in treatment	1	[35]
	Side effects of infertility treatment	1	[35]
	Using donor sperm or eggs	1	[35]
	Surrogacy	1	[35]
	Foster parenting	1	[35]
	Fertility preservation/infertility treatment options	1	[35,39]
**Provider for infertility treatment**		—^a^	—
	Success rates	1	[35,39]
Surrogacy		1	[35]

^a^Not applicable.

**Table 2:** Taxonomy of pregnancy-related information: during pregnancy stage.

Theme and subnodes				N		Source
**Transmittable disease**				2		[24,26]
	STI^a^		2		[24,26]	
**Nutrition, physical exercise, or lifestyle in pregnancy**				9		[10,13,14,22,25,28,29,33,45]
	Nutrition in pregnancy		7		[10,13,14,28,33,40,45]	
	Personal hygiene		2		[33,38]	
	Mother weight		1		[38]	
	Quality of oocyte, embryo, or semen		1		[12]	
	Food safety		2		[24,29]	
	Alcohol consumption during pregnancy		1		[29]	
	Fish and mercury consumption		1		[29]	
	Caffeine consumption		1		[29]	
	Daily activities in pregnancy		4		[28,31,33,38]	
	Sexual activity in pregnancy		2		[13,33]	
**Risk behavior in pregnancy**				1		[10]
	**Maternal complication**		1		[38]	
		Nausea or vomiting	1		[38]	
		Morning sickness	2		[24,38]	
		Maternal fatigue	1		[38]	
		Constipation	1		[38]	
		Heartburn	1		[38]	
		Bloating	1		[38]	
		Lower extremity edema	1		[38]	
		Increased urinary frequency	1		[38]	
		Difficulty sleeping	1		[38]	
		Gestational diabetes	2		[25,31]	
	**Fetal complication**		1		[40]	
		Congenital abnormality	1		[40]	
**Other concerns**				—^b^		—
	Hospital and doctor choices		1		[10]	
	Birth control		1		[24]	
	Environmental exposures		1		[30]	
	Adoption or abortion		3		[24,26,33]	
**Prenatal care**				8		[10,22,24,25,30,33,38,40]
	Coping with minor discomforts during prenatal care		1		[13]	
	Physiology of pregnancy		1		[13]	
	Personal care and hygiene		1		[13]	
	Development, safety of the fetus		5		[10,14,22,25,33]	
	Hospital choices		1		[32]	
	Finding a doctor or midwife		1		[38]	
	**Prenatal diagnostic and screening methods**		1		[40]	
		Fetal echocardiography	1		[40]	
		Amniocentesis	1		[40]	
		Chorionic villus sampling	1		[40]	
		Nuchal translucency scan	1		[40]	
		Blood tests	1		[40]	
		Risks of invasive methods	1		[40]	
		Umbilical cord sampling	1		[40]	
		Fetal magnetic resonance imaging	1		[40]	
		Anatomy scan	1		[11]	
	Issues related to prenatal visits		1		[38]	
	What to expect at prenatal visits		1		[38]	
	Facts about pregnancy		1		[38]	
	Rhogam		1		[38]	
	Dietary supplements during prenatal care		1		[38]	
	Suggestion to wait for labor at or >39 weeks		1		[38]	
	Information about disease in pregnancy		1		[38]	
**Expected body changes and how to handle them**				2		[33,38]
	Effects of pregnancy on mother’s body		1		[33]	
	Effect of pregnancy on work		1		[33]	
**Medication in pregnancy**				6		[13,24,25,28,33,35]
	Side effects		1		[12]	
	Use or application of medication		1		[12]	
	Interaction with other medication		1		[12]	
**Vaccination during pregnancy**				5		[19,25,33,34,38]
	Vaccine safety		1		[34]	
	Pertussis disease		1		[34]	
	Effectiveness of vaccination during pregnancy		1		[34]	
	When to get Tdap^c^		1		[34]	
	Who else should get Tdap		1		[34]	
	Insurance coverage for Tdap		1		[34]	
	Where to get Tdap		1		[34]	
	Cost of Tdap		1		[34]	
**Health care products**				2		[35,38]
	Maternal product safety and product recalls		2		[35,38]	
**Insurance**				1		[38]
	Private health care coverage		1		[35]	
**Labor/delivery**				9		[10,22,24,25,31-33,37,38]
	Methods of delivery		1		[33]	
	**Induction**		1		[46]	
		Indications of induction	1		[46]	
		Oxytocin	1		[46]	
		Cervical ripening	1		[46]	
		Amniotomy	1		[46]	
		Outpatient methods	1		[46]	
		Side effects of induction	1		[46]	
		Failed induction	1		[46]	
	**Preparation for delivery**		1		[10]	
		Common labor fears	1		[38]	
		Pack hospital bag	1		[38]	
		Obtaining a labor doula	1		[38]	
	Mode and process of delivery		1		[10]	
	Delivery stories		1		[10]	
	Due date		1		[24]	
	Peer experience of delivery		1		[32]	
	**Labor pain relief methods**		1		[32,33]	
		Opioids	1		[46]	
		Nitrous oxide	1		[46]	
		Relaxation techniques	1		[38]	
		Spinal anesthesia	1		[46]	
		Continuous labor support	1		[46]	
		Water immersion	1		[46]	
		Sterile water injection	1		[46]	
		Touch and massage	1		[46]	
		Acupuncture and acupressure	1		[46]	
		Hypnosis	1		[46]	
		Transcutaneous electrical stimulation unit	1		[46]	
		Heat and cold	1		[46]	
		Music and audioanalgesia	1		[46]	
		Aromatherapy	1		[46]	
		Biofeedback	1		[46]	
		Labor analgesia	1		[47]	
Oral health				1		[38]
Maternal diet				1		[36]
Diagnostic information				1		[24]
**Miscarriage**				1		[23]
	Causes of miscarriage		1		[23]	
	Frequency of miscarriage		1		[23]	
	Symptoms of miscarriage		1		[23]	
	Research and breakthroughs on miscarriage		1		[23]	

^a^STI: sexually transmitted disease.

^b^Not applicable.

^c^Tdap: tetanus toxoid, reduced diphtheria toxoid, and acellular pertussis.

**Table 3:** Taxonomy of pregnancy-related information: postpartum stage.

Theme and subnodes				N		Source
Maternal recovery				2		[10,32]
Parenting				1		[38]
**Postpartum planning**				1		[38]
	Applying for baby’s social security		1		[38]	
	Newborn jaundice checks		1		[38]	
	Swaddling		1		[38]	
	Nursing pillow		1		[38]	
	Cord blood banking		1		[38]	
**Newborn care**				5		[13,24,33,37,38]
	Finding pediatrician		1		[38]	
	Immunization		1		[40]	
	Side effect of vaccine		1		[34]	
	Newborn developmental milestones		1		[19]	
	Sleep		1		[19]	
	Fever		1		[19]	
	Outdoors		1		[19]	
	Stooling		1		[19]	
	Newborn coughs and colds		1		[19]	
	Newborn gas and burping		1		[19]	
	Newborn bathing		1		[19]	
	Newborn circumcision		1		[19]	
	Newborn growth		1		[19]	
	Pacifier		1		[19]	
	Teething		1		[19]	
	Neonatal complications		1		[32]	
	Health issues of newborn		1		[24]	
	**Milk feeding**		3		[13,19,24,27,31,33,36-38]	
		Bottle feeding	1		[27]	
		Breastfeeding	8		[19,24,27,31,36-38]	
		Continued breastfeeding	1		[36]	
		Breast milk substitutes	1		[36]	
		Formula feeding	1		[19]	
	**Solid food**		1		[36]	
		Solid foods–timing	1		[36]	
		First foods to introduce	2		[19,36]	
		Foods to avoid	1		[36]	
		Food allergens	1		[36]	
		Spacing of new foods	2		[10,45]	
**Postpartum care**				1		[33]
	Physical and psychological complications after delivery		1		[33]	
	Self-care after birth		1		[13]	
**Miscarriage**				1		[23]
	Causes of miscarriage		1		[23]	
	Frequency of miscarriage		1		[23]	
	Symptoms of miscarriage		1		[23]	
	Research and breakthroughs on miscarriage		1		[23]	

**Table 4:** Taxonomy of pregnancy-related information: all-stage.

Theme and subnode		N	Source
**Test**		6	[11,24,32,33,35,40]
	Special tests during pregnancy	1	[33]
	DNA testing	1	[24]
	Interpreting results of diagnostic tests	1	[35]
	Blood sugar test	1	[31]
Family planning		1	[33]
Planning for a newborn		1	[38]
Preparing for a new pregnancy		1	[23]
**Mental health**		4	[24,27,33,38]
	Preexisting or antepartum/postpartum anxiety or depression	1	[38]
	Seeking support with friends, family, your doctor	1	[38]
	Emotions in pregnancy	1	[38]
	Establishing support network, including checking in with a friend, reaching out to other moms	1	[38]
	Stress management	1	[38]
	Relaxation techniques	1	[38]
	Management	1	[38]
	Genetic screening, amniocentesis, or chorionic villus sampling	1	[38]
	Ultrasounds	1	[38]

### Co-Occurrence of Consumer Information Needs

**Figure 3:**
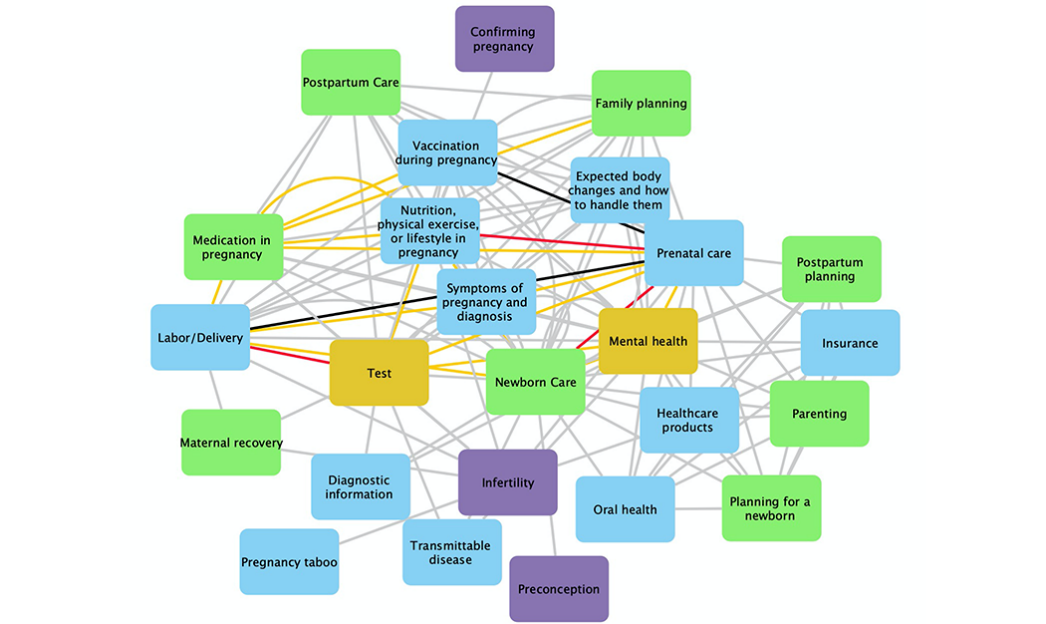
Network graph of co-occurring information needs in articles.

Figure 3 presents the network graph for the co-occurrence of consumer information needs in all 33 articles. Purple, blue, green, and yellow nodes refer to prepregnancy, during pregnancy, postpartum, and multistage, respectively. Gray, orange, red, and black edges refer to the co-occurrence of 1, 2, 3, and 4 times, respectively. For example, consumer information needs related to infertility co-occur with medication in pregnancy, health care products, lab test, nutrition, physical exercise, or lifestyle in pregnancy, pregnancy taboo, prenatal care, labor/delivery, and maternal recovery once, respectively. Consumer information needs about labor/delivery co-occur with maternal recovery once, symptoms of pregnancy and diagnosis twice, test 3 times, and prenatal care 4 times. Consumer information needs about family planning co-occur with newborn care, postpartum care, and mental health once, respectively. Consumer information needs about mental health co-occur with health care products, insurance, oral health, parenting, planning for a newborn, and postpartum planning, respectively.

### Intersection of Consumer Information Needs and Countries of Participants

**Figure 4:**
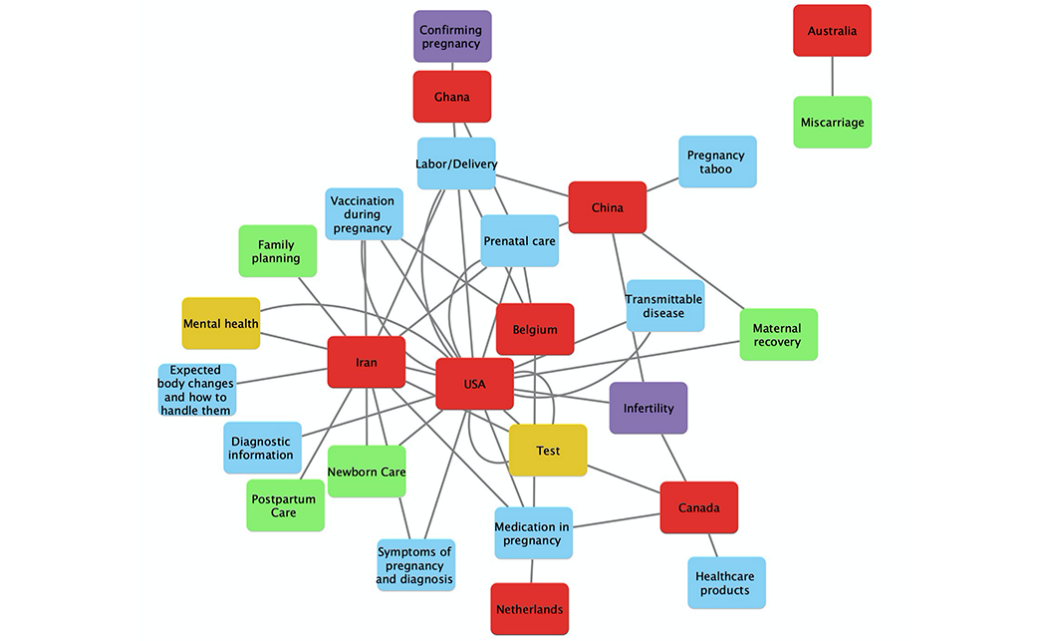
Network graph of information needs in countries of participants.

For all human-subject studies in this review, Figure 4 presents the network graph for intersections between consumer information needs and countries of participants. For example, participants in Ghana sought information related to confirming pregnancy, labor/delivery, nutrition, physical exercise, and lifestyle in pregnancy, while information about medication in pregnancy was sought by participants in Belgium, Canada, Iran, Netherlands, and the United States. This figure only represents the demographics of needs of participants by country in all reviewed articles and could not be generalized to describe the needs of consumers in each of these countries.

## Discussion

### Summary

Pregnancy is a major life-changing event and is considered one of the most sensitive periods of a women’s life. Special care and various types of information are required during this period. However, due to reasons like health literacy and technology proficiency [32,37,38], women feel pregnancy-related information is insufficient and not well organized. Therefore, it is important to organize such information in a systematic way to meet the consumer information needs of this population. In this study, we assessed pregnancy-related consumer information needs and available information from different sources through the review of relevant articles. To the best of our knowledge, this is the first study to systematically assess consumers’ pregnancy-related consumer information needs across all stages of the pregnancy trajectory. It is also the first study to derive a taxonomy of pregnancy-related information through a systematic literature review. Existing taxonomies of pregnancy-related needs in the included articles mainly focused on one or two stages of pregnancy [10,13,21,22,24,27,30,33,41] or a facet of pregnancy (eg, female infertility [12], nutrition [28,29,42], physical activity [28,48], vaccines [34], medications [25], labor analgesia [47], congenital heart defects [40], infant feeding [36], or miscarriage [23]). Our study systematically assessed consumer information needs and sources across the entire pregnancy trajectory, including preconception, during pregnancy, and postpartum.

In the last 50 years, the role of the patient has changed. Previously medical professionals were seen as the sole decision makers and information providers. Now patients are more responsible for decision making and personal information seeking. Given the nature of pregnancy decisions, this has definitely impacted this population. Consumers’ online information seeking about pregnancy could be to obtain a sense of empowerment and preparedness in interacting with health care providers [49]. They also need to be able to understand the information presented by health care providers and better monitor decisions made with doctors [49]. High-quality information on the internet is often appreciated by consumers so that they can also use this information for decision making [50].

There are many theories that have been developed to provide a framework to examine information behaviors. One theory that applies to pregnancy information seeking is Dervin’s sense-making theory [51]. The goal of sense-making may not always be finding relevant information but may include finding people in similar situations and avoiding bad information. Sense-making is not new and not limited to the information fields. It has also been seen in organization, communication, education, and human-computer interaction. In health care, it has been used in diabetes management [52], tumor contouring [53], and Down syndrome diagnosis [54]. One premise of this theory is the connection between how someone looks at a situation and how they are able to understand or make sense of this situation. Think about the gap between what you know and what you need or want to know. In pregnancy, this gap is almost guaranteed even in individuals who have had successful pregnancies in the past. This gap can be influenced by many personal factors including history, previous education, adaptability, and skill at building connections with information. Ultimately, these factors are more individual and personal then situational. This moves the assumptions that are made from the individual to the instances of information seeking. This allows for studies like this to focus on the differences in the information and group the population together even though there are many differences in this population.

Traditional information sources (eg, health care providers) often provide critical and general information about pregnancy. However, such information is often not contextualized in personal situations. In addition, the medical jargon used in the information presented in traditional sources makes it difficult for consumers to understand [55]. As a result, the internet has become an alternative source of information. During the online information-seeking process, health literacy plays an important role. Prior studies found that consumers with low health literacy tend to use the internet less frequently [56,57] and find it challenging to retrieve and appraise online health information about pregnancy, which could in turn impair their ability in decision making and achieving better pregnancy outcomes [58]. Furthermore, our study found that only 2 out of the 33 articles about consumer information needs and sources assessed the barrier to accessing pregnancy-related information online and no article investigated the impact of health literacy on consumer information needs related to pregnancy. Therefore, future work should carefully examine consumers’ health literacy so that information can be provided to consumers based on their different levels of health literacy.

We also found that most studies in this review assessed consumers’ pregnancy-related information needs and the available information from different sources. However, only a few studies examined the quality of the information provided by these sources, which is consistent with the finding of the previous review [14]. Among the information quality criteria, readability was one of the most frequently cited issues [59]. Berland et al [7] found that English and Spanish websites required high levels of reading ability. Also, consumers often find that online health information contains a high level of technicality with lots of medical jargon [60]. To make better use of the internet, the readability of the content should be presented at or below a 5th grade reading level to accommodate people of all health literacy levels [61]. Last, the booming production of online health information has resulted in information overload [62]. To address these issues, it is important that health care providers and supportive technologies develop ways to direct consumers to high-quality sources that are layperson-friendly and pertinent to their situation.

In this review, we also intended to identify the types of information that are needed by consumers but have not been provided by different sources including websites, mobile apps, and health care providers. However, there are a few reasons why this goal could not be achieved. First, most of the articles are about consumer information needs but only a limited number of articles are about information sources. Also, the focus and ways of organizing information in these articles are different. For example, some articles focused on a specific stage of pregnancy while others focused on a specific disease or complication related to pregnancy, and thus there were not many common themes of information provided. Second, the included articles about information sources could not represent the comprehensive types of information available in different platforms. Hence, future work can consider using systematic approaches to assess themes of information about pregnancy available on all the different platforms.

In addition, our study resulted in a taxonomy of pregnancy-related information. This taxonomy can be implemented into supportive technologies (eg, webpages, smartphone apps, and patient portals) so that consumers can easily access and retrieve a structured body of information about pregnancy regardless of the stage of their pregnancy. We have uploaded our PINO taxonomy to our GitHub repository [63] and BioPortal [64]. Currently, PINO only includes 267 classes and 271 hierarchical relationships. In the future, we will improve PINO by adding more concepts and semantic relationships between concepts to further express the conceptual domain space across all stages of pregnancy—prepregnancy, during pregnancy, and postpartum.

### Research Gaps

The result of this review suggested several research gaps. First, only a few studies assessed information quality of the sources of pregnancy-related information. Furthermore, these studies suggested that the quality of the provided information is questionable. Given that prior work has proposed a model for assessing consumer health information quality [65], future work can systematically extract and assess the quality of pregnancy-related information from all available sources (eg, providers’ webpages, online health forums, mobile apps).

In addition, this review suggests that very little is known about how much consumer information needs about pregnancy have been satisfied and what needs are not yet met. Future work can consider comprehensively examining both consumer satisfaction and unmet consumer information needs about pregnancy.

Last, given the overwhelming amount of available health information online and the filter failure of existing information retrieval systems [62], future work could explore ways to help consumers retrieve high-quality, customized, and layperson-friendly health information.

### Limitations

This review has limitations. First, we only systematically assessed articles about pregnancy-related consumer information needs and sources published from 2009 to 2019. Second, articles in this study have different focuses and organization of pregnancy-related information themes. Neither consumer information needs nor available information could be generalized to all pregnant women or sources. Hence, conclusions regarding which consumer information needs have been met could not be made.

### Conclusions

In this study, we reviewed 33 articles published from 2009 to 2019 about pregnancy-related consumer information needs and available information from different sources. The resulting taxonomy comprehensively covered and provided hierarchal themes of pregnancy-related consumer information needs across the stages of pregnancy. Last, findings of this study suggested several future research directions: systematically assessing the quality of pregnancy-related information from all available sources, comprehensively examining both consumer satisfaction and unmet consumer information needs about pregnancy, and exploring ways to help consumers retrieve high-quality, customized, and layperson-friendly health information.

## Appendix

Multimedia Appendix 1 Detailed information about the Critical Appraisal Skills Program (CASP) review of the included papers.

Multimedia Appendix 2 Characteristics of the included papers.

## Abbreviations

CASP: Critical Appraisal Skills Program
PINO: Pregnancy Information Needs Ontology
PRISMA: Preferred Reporting Items for Systematic Review and Meta-analysis
