# Supplementary material for: Understanding Information Needs and Barriers to Accessing Health Information Across All Stages of Pregnancy: Systematic Review
Source: JMIR Pediatr Parent. 2022 Feb 21;5(1):e32235. doi: 10.2196/32235 (PMC8902674; doi:10.2196/32235)
Supplement: Multimedia Appendix 2 [file pediatrics_v5i1e32235_app2.pdf]

Multimedia Appendix 2

Table 1. Characteristics of all included articles

Citation	Topic	Method of Data Collection	Country of Participants	Sample Size	Health Information Source	Stage	Target Population
Linden (2012) [31]	Web-based information for pregnant women and new mothers with type 1 diabetes	Participatory design & evaluation	N/A	7	Internet	During pregnancy & Postpartum	Pregnant women and new mothers with type 1 diabetes
Song (2013) [24]	Information needs, seeking behavior, and support of low-income expectant women	Survey/Questionnaire	USA	63	NA	During pregnancy	Expectant women
Van De Belt (2014) [12]	Gaps in information sources for infertile patients	Secondary data	Netherlands	NA	Healthcare providers	Pre-pregnancy	Dutch infertile patients who received one of the following fertility treatments: IUI, IVF, ICSI or OI
Carlsson (2015) [40]	Information websites about congenital heart defects following a prenatal diagnosis	Secondary data	NA	67	Internet	During pregnancy	Websites about congenital heart defects
Owusu-Addo (2016) [22]	Health information-seeking behaviours among pregnant teenagers in Ejisu-Juaben Municipality, Ghana	interview	Ghana	28	NA	During pregnancy	Pregnant teenagers
Guerra-Reyes (2016) [27]	Low-income mothers' perceptions of their postpartum information needs	Survey & Interview	USA	10	Mobile applications	Postpartum	Low-income postpartum women

O'Donnell (2016) [38]	Informative content of two free, pregnancy-specific smartphone applications and their accuracy and adherence to prenatal care guidelines	Secondary data	NA	609 (pieces of content)	Mobile applications	Multiple stages	Pregnancy-specific smartphone applications
Narasimhulu (2016) [32]	Patterns of e-health use in pregnancy in an underserved racially diverse inner-city population & Reliability of pregnancy-related searches]	Survey/Questionnaire	USA	503	Internet	During pregnancy & Postpartum	Pregnant women and postpartum women
Sayakhot (2016) [14]	Internet use by pregnant women seeking pregnancy-related information	Systematic literature Review	NA	7	Internet	During pregnancy	Pregnant women
Netting and Allen (2017) [36]	Infant feeding for allergy prevention	Secondary data	NA	25	Internet	Postpartum	Australian consumers
Storr (2017) [42]	Pregnancy-related food and nutrition information available online	Secondary data	NA	693	Internet	During pregnancy	Websites about pregnancy nutrition information
Boztas (2017) [47]	Readability of internet-sourced patient education material related to “labour analgesia”	Secondary data	NA	38	Internet	During pregnancy & Postpartum	Internet-sourced patient education material related to “labour analgesia”
Rotich and Wolvaardt (2017) [21]	Health information needs of Kenyan women in the first 6 weeks postpartum	interview	Kenya	15	NA	Postpartum	Kenyan women in the first 6 weeks postpartum

Guerra-Reyes (2017) [41]	Disconnection between postpartum health information desired and health information received	Survey	USA	77	Healthcare providers & Family & Friends & Internet	Postpartum	Mothers of young children (age 48 months or younger) in Monroe County, Indiana.
Artieta-Pinedo (2018) [37]	Online information sources regarding pregnancy, birth and the postnatal period	Secondary data	N/A	126	Internet	During pregnancy & postpartum	Spanish and English on-line information sources
English (2018) [46]	Content in websites about induction of labor and pain management	Secondary data	N/A	11	Internet	During pregnancy	Websites about induction of labor and pain management
de Man (2018) [39]	availability of web content on fertility preservation for young women facing cancer	Secondary data	N/A	33	Internet	Pre-pregnancy	Websites about fertility preservation for young women facing cancer
Kamali (2018) [33]	Information needs of pregnant women during their pregnancy and childbirth	Survey/Questionnaire	Iran	400	Healthcare providers	During pregnancy	Women in Kerman, Iran who had an academic degree
Pang (2018) [23]	Online information - seeking behavior of women experiencing miscarriage	interview	Australia	12	Internet	Postpartum	Women who experience miscarriage
Robinson (2018) [11]	Health-related needs of pregnant women and their caregivers	Survey & Interview	USA	71 pregnant women and 29 caregivers	Internet	During pregnancy	Pregnant women and their caregivers
Kallem (2018) [19]	Low-income mothers' information needs about infant	Randomized controlled trial	USA	43	NA	Postpartum	Low-income mothers
Holton (2018) [20]	Fertility concerns and related information needs and preferences of women with PCOS	Secondary data	N/A	N/A	Internet	Pre-pregnancy	Women with PCOS

Cramer (2018) [26]	Expectant and recent (E/R) fathers' health information behavior during pregnancy, childbirth, and childcare.	Survey/Questionnaire	USA	186	NA	During pregnancy & postpartum	Expectant and recent (E/R) fathers
Uhm and Choi (2019) [45]	Mothers' needs in forming partnerships with nurses based on children's postoperative recovery in a paediatric cardiac intensive care unit	Secondary data	NA	36	NA	Postpartum	New mothers
Arcia (2019) [30]	Information needs and information-seeking process of low-income pregnant women	Focus Group	USA	16	NA	During pregnancy	Low-income pregnant women
Zhu (2019) [10]	Pregnancy-related information seeking and sharing via social media among Chinese expectant mothers	Interview	China	20	Internet	During pregnancy & Postpartum	Chinese expectant mothers
Harrison (2019) [48]	Women's attitudes and perceptions of barriers and enablers to physical activity in pregnancy	Interview	Australia	27	Health care providers & Internet	During pregnancy	Women with gestational diabetes mellitus
Kriss (2019) [34]	Vaccine Information Needs Among Pregnant Women in the United States	Survey/Questionnaire	USA	486	NA	During pregnancy	Pregnant Women in the United States
Brochu (2019) [35]	What individual factors are associated with searching the internet for infertility-related information, and whether Web-based resources meet the needs of patients	Survey/Questionnaire	Canada	567	Internet	Pre-Pregnancy	Men and women seeking infertility care

Ceulemans (2019) [25]	Beliefs about medicines and information needs among pregnant women	Survey/Questionnaire	Belgium	372	Internet & Healthcare providers	During pregnancy	Pregnant women visiting a tertiary hospital in Belgium
Cannon (2020) [28]	Pregnancy information on nutrition, physical activity and sleep	Systematic Literature Review	N/A	27	Internet	During pregnancy & postpartum	Pregnancy information on nutrition, physical activity and sleep websites
Brown (2019) [29]	Quality, inclusion of behaviour change techniques, and nutrition information of pregnancy iPhone apps	Systematic literature Review	N/A	51	Mobile applications	During pregnancy & postpartum	Apps in Australian iTunes app store
Ghiasi (2019) [13]	Health information needs, sources of information and barriers to accessing of women during pregnancy	Systematic literature Review	N/A	31	Healthcare providers & Family & Friends & Internet	During pregnancy	Pregnant women
